# Multi-Omics Reveals Tetrodotoxin Transport and Accumulation Mechanisms in *Takifugu bimaculatus*

**DOI:** 10.3390/md24050172

**Published:** 2026-05-10

**Authors:** Xinxin Zhang, Min Xu, Jiapeng Qi, Shuigen Li, Xiaoting Chen, Bei Chen, Shuilin Cai, Kun Qiao, Qilin Huang, Zhiyu Liu

**Affiliations:** 1 College of Food Science and Technology, Huazhong Agricultural University, Wuhan 430070, China; 18764245595@163.com; 2 Fisheries Research Institute of Fujian, Key Laboratory of Cultivation and High-Value Utilization of Marine Organisms in Fujian Province, National Research and Development Center for Marine Fish Processing, Xiamen 361021, China; 3 Fisheries College, Jimei University, Xiamen 361021, China; 4 Fujian Fisheries Technology Extension Center, Fuzhou 350028, China

**Keywords:** *Takifugu bimaculatus*, tetrodotoxin, transport, accumulation, transcriptomics, proteomics

## Abstract

The potent toxicity of tetrodotoxin (TTX) has long constrained sustainable growth in pufferfish aquaculture. *Takifugu bimaculatus*, an economically important species farmed along the coast of Fujian, China, remains poorly understood regarding how it transports and accumulates this potent neurotoxin. To address this gap, we combined transcriptomic and proteomic analyses to characterize the molecular responses of *T. bimaculatus* to TTX exposure. After oral administration, TTX primarily accumulated in the liver, ovaries, and skin. Multi-omics profiling revealed 163 differentially expressed genes (DEGs) and 88 differentially expressed proteins (DEPs) in liver tissue, together with 239 DEGs and 179 DEPs in ovarian tissue. KEGG pathway analysis suggests that the liver maintains homeostasis by regulating ion concentrations and restructuring lipid raft architectures, alongside coordinated carrier protein activity. This likely supports active TTX uptake and directed transport toward the ovaries and skin, followed by metabolic clearance. By contrast, ovarian tissues appear to establish a stable, long-term reservoir through cytoskeletal remodeling, enhanced interactions with the extracellular matrix, and activated endocytic pathways. Together, these findings offer insights into how *T. bimaculatus* accumulates and transports TTX, laying groundwork for identifying key transporter genes, clarifying TTX metabolic pathways, and developing practical food safety controls.

## 1. Introduction

Tetrodotoxin is a potent, low-molecular-weight neurotoxin that occurs naturally in diverse taxa, having first been identified in pufferfish [1,2] and subsequently detected in crustaceans, gastropods, bivalves, nemerteans, planocera and amphibians [3,4,5,6,7]. In human poisoning, the toxin blocks nerve transmission by binding to voltage-gated sodium channel receptors, causing progressive muscular paralysis, and in severe cases, respiratory and cardiac failure [8,9]. Its origins remain debated: some evidence points to endogenous production by symbiotic bacteria such as *Pseudomonas* and *Vibrio* spp., while other data support exogenous accumulation through trophic transfer and biomagnification within food webs [10,11]. Notably, TTX withstands conventional cooking temperatures, making it particularly hazardous as a foodborne threat [12]. Fatalities from pufferfish poisoning continue to occur worldwide, underscoring the persistent public health challenge [13,14,15].

How pufferfish transport and accumulate TTX has gradually become clearer through recent work. Yamamori et al. [16] showed that non-toxic *T. rubripes* and grass pufferfish (*T. niphobles*) rapidly shuttle ingested TTX via the bloodstream to target organs, particularly the liver and ovaries. Tatsuno et al. [17] extended these findings by tracking age-dependent distribution patterns in cultured *T. rubripes*: juveniles (6 months) stored most TTX in the skin, whereas adults (15 months) showed preferential hepatic sequestration. Sex differences also emerge prominently, with ovarian concentrations far exceeding testicular levels—a pattern suggesting estrogen-mediated regulation of toxin storage [18,19].

*Takifugu bimaculatus*, an economically important species farmed along the coast of Fujian, China, offers an ideal model for probing these dynamics, yet how this species specifically transports and accumulates TTX remains unclear. We therefore conducted controlled oral gavage experiments to track spatiotemporal changes in tissue TTX concentrations, pairing these measurements with integrative transcriptomic and proteomic analyses of liver and ovarian tissues from control (phosphate-buffered saline [PBS]) and TTX-treated groups. Gene Ontology (GO) and Kyoto Encyclopedia of Genes and Genomes (KEGG) pathway analyses helped us piece together the molecular machinery governing TTX transport and tissue-specific accumulation. Beyond advancing our understanding of tetrodotoxin metabolism, these results should inform practical strategies for monitoring and mitigating food safety risks in aquaculture settings.

## 2. Results

### 2.1. Tissue-Specific Distribution of TTX

**Figure 1:**
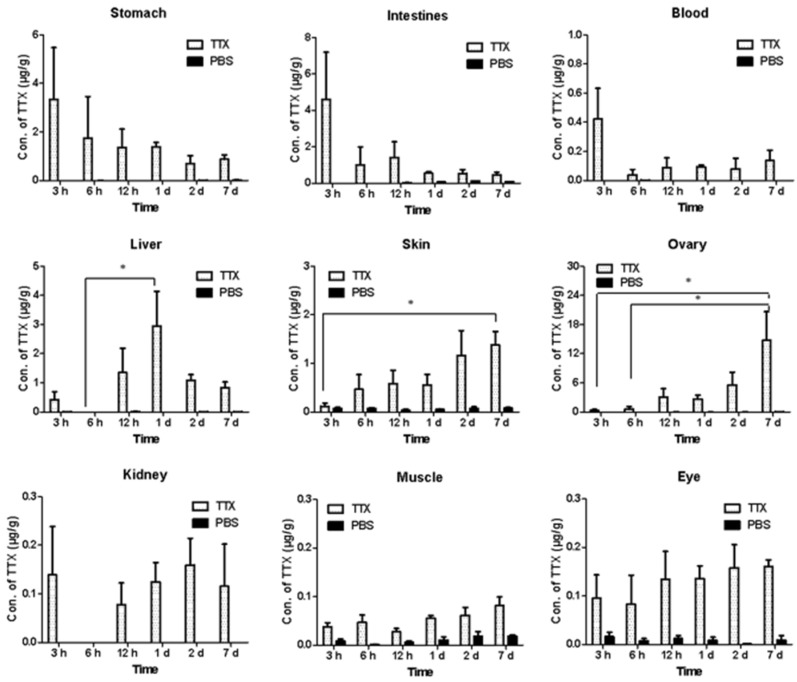
Distribution of TTX levels in various tissues of *Takifugu bimaculatus*. * *p* < 0.05. (TTX: TTX-treated group; PBS: PBS-treated group).

This study followed the provisions of GB 5009.206-2016 [20] “National Food Safety Standard: Determination of Tetrodotoxin in Aquatic Products,” as well as regulations from Japan’s Ministry of Health, Labor and Welfare and the Local Standard of Fujian Province, China. Fresh food with TTX < 2.2 μg/g is considered non-toxic, with 2.2 μg/g < TTX < 22 μg/g defined as weakly toxic. Using this as a reference, the dynamic changes in TTX concentrations in the stomach, intestine, plasma, liver, skin, ovaries, kidneys, eyes, and muscle tissues were systematically detected (Figure 1). In the experiment, the control group administered PBS via gavage did not test positive for TTX. Gastrointestinal toxin levels peaked at 3 h after TTX administration, then declined from a weakly toxic level to a non-toxic level. TTX levels in the blood also peaked at 3 h after administration and subsequently dropped rapidly. TTX levels in liver tissue rose gradually after administration, reaching a weakly toxic level (2.25 μg/g) by 1 day, showing a significant difference compared to levels at 6 h post-administration (*p* < 0.05); and subsequently decreased rapidly, returning to non-toxic levels. In ovarian tissue, TTX levels showed a continuous upward trend after gavage; by day 7, TTX levels had approached highly toxic levels (14.73 μg/g), representing 42 times the level at 3 h post-gavage (*p* < 0.05) and 25 times the level at 6 h post-gavage (*p* < 0.05). This indicates that ovarian tissue has a stronger toxin accumulation capacity than other tissues. TTX levels in the skin also showed a continuous upward trend, rising from less than 0.17 μg/g at 3 h post-administration to nearly 1.38 μg/g at 168 h (*p* < 0.05); however, the accumulation rate was slower, and levels remained within the non-toxic range. Following administration, TTX levels in the kidneys and eyes did not change significantly over time, remaining stable at non-toxic levels. TTX levels in muscle were the lowest among all tissues, showing a slight upward trend but no significant difference, and remained at non-toxic levels throughout.

### 2.2. Characteristics of Transcriptome Responses Following TTX Exposure

#### 2.2.1. Construction and Quality Control of Transcriptome Libraries from the Liver and Ovaries of *Takifugu bimaculatus* Under TTX Exposure

**Figure 2:**
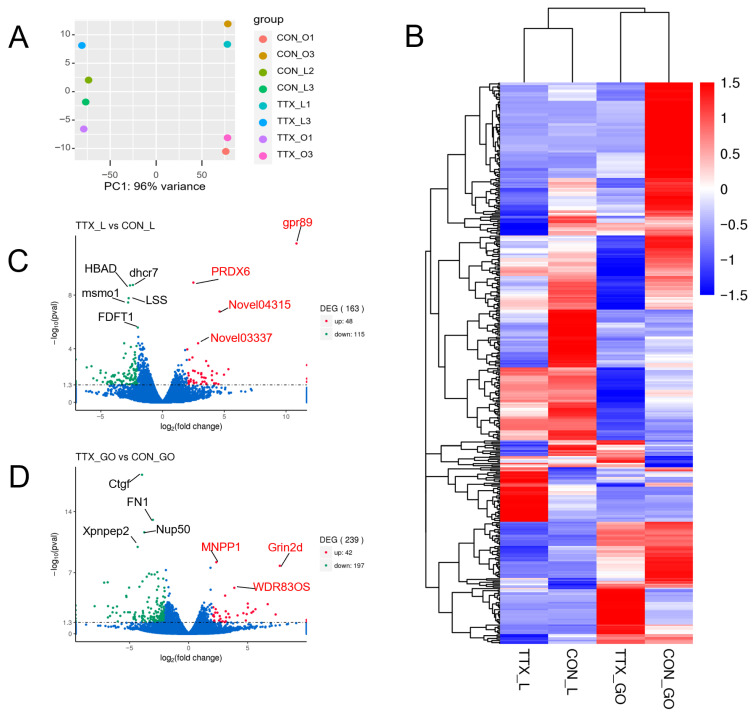
Transcriptomic analysis of liver and ovarian tissues from *Takifugu bimaculatus* following TTX gavage. (**A**) Principal component analysis; (**B**) Clustering heatmap using average values; (**C**) Volcano plot of liver tissue; (**D**) Volcano plot of ovarian tissue. CON_L: The liver control group; TTX_L: The liver TTX group; CON_O/CON_GO: The ovaries control group; TTX_O/TTX_GO: The ovaries TTX group.

A statistical table showing the alignment of reads to the *Takifugu bimaculatus* genome is provided in Supplementary Table S1. Figure 2A,B depict the differentially expressed transcriptomic profiles between the TTX and control groups in liver and ovarian tissues.

The input data for differential gene expression analysis consisted of read count data obtained from gene expression profiling. The DE-Seq software (1.16.1) was used to normalize the read counts, and *p*-values were calculated based on a negative binomial distribution model. The screening criteria for differentially expressed genes (DEGs) were *p* < 0.05 and |log_2_ fold change| > 2. A total of 163 differentially expressed genes were identified in the liver of *Takifugu bimaculatus*, including 48 upregulated and 115 downregulated genes (Figure 2C). A total of 239 differentially expressed genes were identified in the ovaries of *Takifugu bimaculatus*, including 42 upregulated and 197 downregulated genes (Figure 2D).

#### 2.2.2. Validation of Differentially Expressed Genes by RT-qPCR

**Figure 3:**
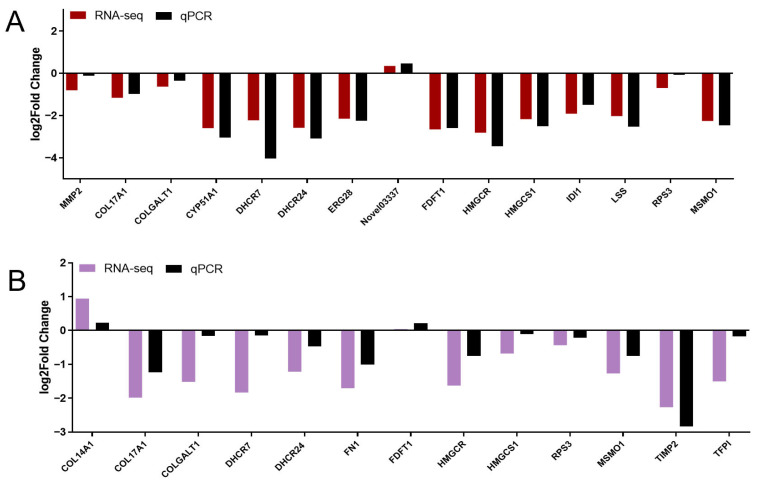
Validation of RNA-seq results by RT-qPCR. (**A**) RT-qPCR validation of differentially expressed genes in liver tissue; (**B**) RT-qPCR validation of differentially expressed genes in ovarian tissue.

The gene expression levels determined by RT-qPCR were consistent with the RNA-seq results (Figure 3A,B), confirming the reliability of the transcriptomic findings.

#### 2.2.3. Enrichment Analysis of Differentially Expressed Genes in the Liver of *Takifugu bimaculatus* Following TTX Exposure

**Figure 4:**
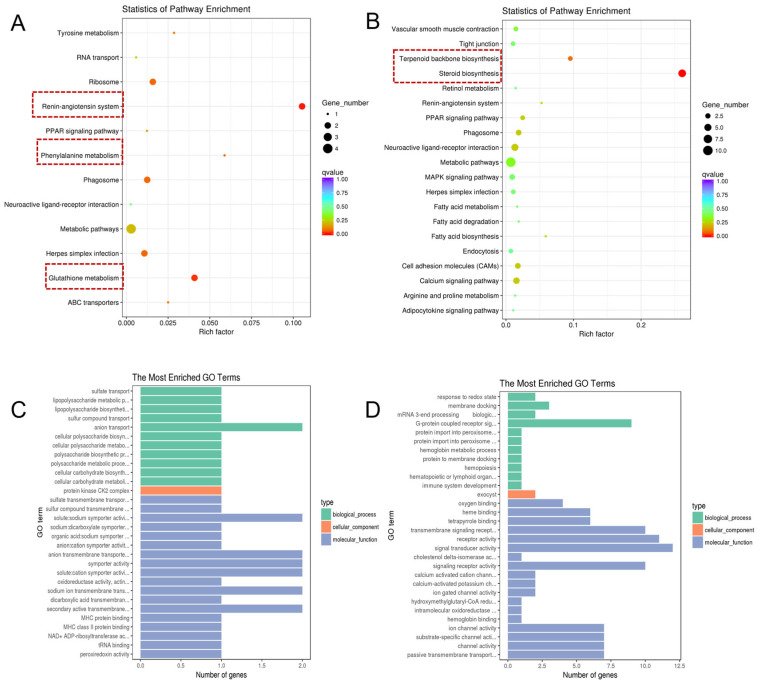
Gene enrichment analysis of liver tissues from *Takifugu bimaculatus* following TTX gavage. (**A**) KEGG enrichment analysis of up-DEGs in the liver; (**B**) KEGG enrichment analysis of down-DEGs in the liver; (**C**) GO enrichment analysis of up-DEGs in the liver; (**D**) GO enrichment analysis of down-DEGs in the liver. up-DEGs: upregulated differentially expressed genes, down-DEGs: downregulated differentially expressed genes. The red boxes: Key pathway of interest. Full names of the GO terms are listed in Supplementary Table S3.

To elucidate the regulatory mechanisms involved in TTX transport and accumulation within the pufferfish, this study performed KEGG pathway enrichment analysis and GO functional annotation analysis on liver DEGs (Figure 4).

KEGG pathway enrichment results showed that upregulated differentially expressed genes (up-DEGs) were significantly enriched in pathways such as the renin-angiotensin system, phenylalanine metabolism, and glutathione metabolism (Figure 4A). Downregulated genes (down-DEGs) were significantly enriched in pathways related to steroid biosynthesis and terpenoid skeleton biosynthesis (Figure 4B).

GO functional annotation analysis (Figure 4C,D, showing the top 30 enriched terms, *p* < 0.05) revealed that up-DEGs associated with biological processes were primarily involved in the biosynthesis and metabolism of lipopolysaccharides, the transport of sulfides/sulfates, and the biosynthesis and metabolism of cellular polysaccharides and carbohydrates; up-DEGs related to cellular composition were primarily involved in the protein kinase CK2 complex; up-DEGs related to molecular function were primarily involved in transmembrane transport processes across multiple pathways, including the transport of sulfides, sodium ions, dicarboxylic acids, and secondary active transmembrane transport, and were also associated with the activity of various transporters or proteins such as sodium-dicarboxylate transporters, organic acid: sodium carriers, cotransporters, MHC proteins, and other carriers or protein activities. Down-DEGs related to biological processes primarily involve membrane docking, signaling of G protein-coupled receptors, protein import into peroxisomes, and hemoglobin metabolism; down-DEGs related to cellular composition primarily participate in exosome vesicle formation. Downregulated DEGs related to molecular function primarily involve oxygen/heme binding, receptor activity, signal transduction activity, calcium-activated cation channel activity, and intramolecular oxidoreductase activity.

#### 2.2.4. Enrichment Analysis of Differentially Expressed Genes in the Ovaries of *Takifugu bimaculatus* Under TTX Exposure

**Figure 5:**
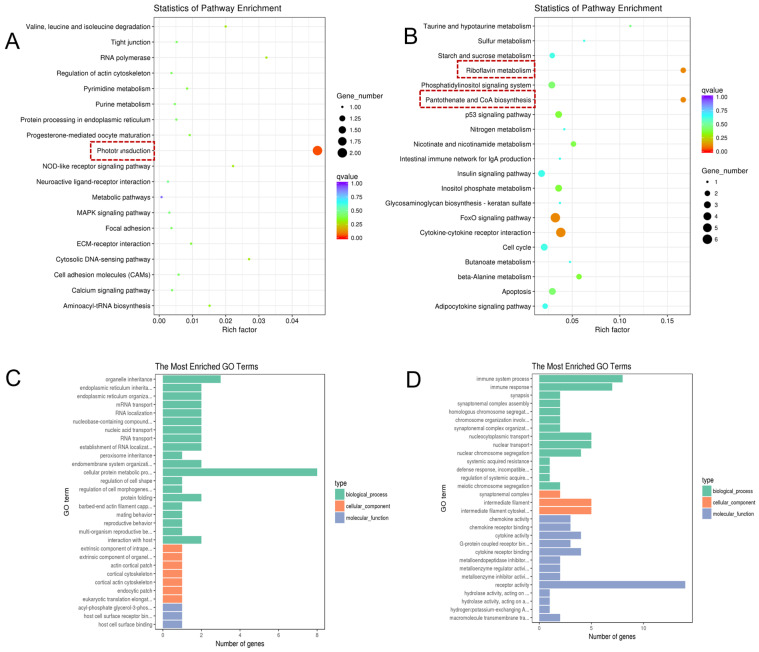
Gene enrichment analysis of differentially expressed genes in the ovaries of *Takifugu bimaculatus* following TTX gavage. (**A**) KEGG enrichment analysis of up-DEGs in the ovaries; (**B**) KEGG enrichment analysis of down-DEGs in the ovaries; (**C**) GO enrichment analysis of up-DEGs in the ovaries; (**D**) GO enrichment analysis of down-DEGs in the ovaries. up-DEGs: upregulated differentially expressed genes, down-DEGs: downregulated differentially expressed genes. The red boxes: Key pathway of interest. Full names of the GO terms are listed in Supplementary Table S4.

The KEGG pathway enrichment results for ovarian DEGs (Figure 5A,B) show that up-DEGs were significantly enriched in the phototransduction pathway, while down-DEGs were primarily enriched in the riboflavin metabolism, pantothenic acid, and coenzyme A biosynthesis pathways.

GO functional annotation analysis (Figure 5C,D, showing the top 30 enriched terms, *p* < 0.05) revealed that up-DEGs associated with biological processes were primarily involved in RNA transport and localization, cellular protein metabolism, and reproductive behavior. Up-DEGs associated with cellular components were mainly involved in the external components of the endoplasmic reticulum and organelles, the cortical/cortical actin cytoskeleton, and the formation of endosomal vesicles; up-DEGs related to molecular function primarily exhibit acyl-3-phosphoglycerate-3-phosphate acyltransferase activity and participate in host cell surface receptor binding. Down-DEGs related to biological processes mainly involve immune response, homologous chromosome separation, and meiosis; down-DEGs associated with cellular composition primarily participate in the synaptonemal complex and the intermediate filament cytoskeleton; down-DEGs associated with molecular function primarily exhibit chemokine activity, cytokine activity, metalloproteinase regulator/inhibitor activity, and participate in receptor binding (chemokine receptors, G protein-coupled receptors, cytokine receptors), among other functions.

### 2.3. Proteomic Response Characteristics Following TTX Exposure

#### 2.3.1. Proteomic Analysis of the Liver in *Takifugu bimaculatus* Following TTX Exposure

**Figure 6:**
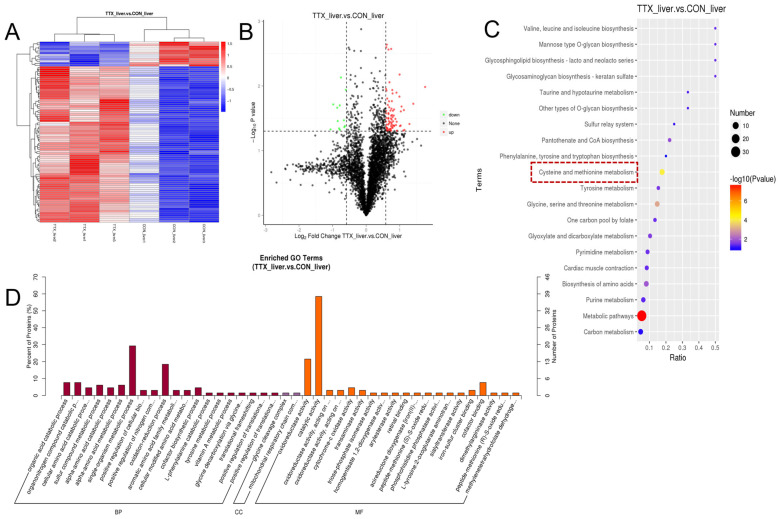
Differential proteomics analysis of liver tissue from *Takifugu bimaculatus* following TTX gavage: (**A**) Clustering heatmap; (**B**) Volcano plot; (**C**) KEGG enrichment analysis of DEPs; The red boxes: Key pathway of interest. (**D**) GO enrichment analysis of DEPs. DEPs: Differentially expressed proteins. Rad: Biological Process (BP); Purple: Cellular Component (CC); Orange: Molecular Function (MF). Full names of the GO terms are listed in Supplementary Table S5.

As shown in the clustering heatmap in Figure 6A, there were significant expression differences between the control and TTX groups in liver tissue, and the samples within each group exhibited good reproducibility. The volcano plot illustrates the distribution of differentially expressed proteins (DEPs) between the two groups. A total of 88 significantly altered proteins were identified, including 76 upregulated and 12 downregulated proteins (Figure 6B). Subsequent enrichment analysis of the DEPs (Figure 6C,D) revealed that they were involved in the composition of the glycine cleavage complex and the mitochondrial respiratory chain complex and were associated with functions such as redox enzyme activity and catalytic enzyme activity. KEGG enrichment analysis further indicated that DEPs were primarily involved in cysteine and methionine metabolism. These results suggest that amino acid metabolic pathways play a crucial role in TTX transport and accumulation in the liver tissue of *Takifugu bimaculatus*.

#### 2.3.2. Proteomic Analysis of the Ovaries of *Takifugu bimaculatus* Under TTX Exposure

**Figure 7:**
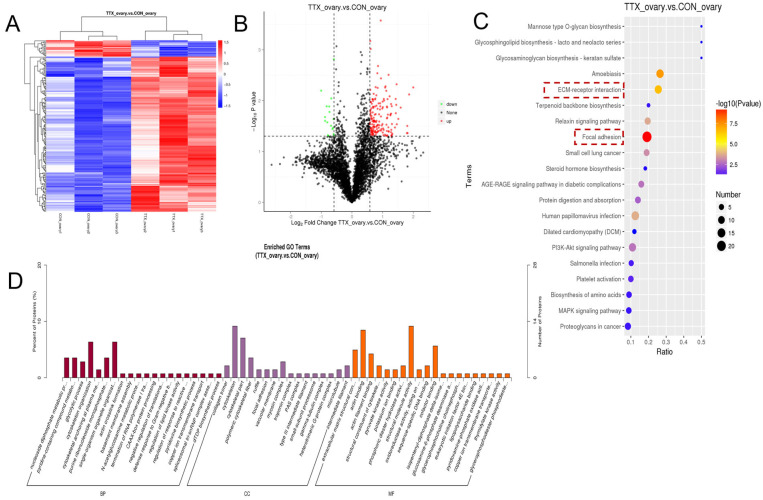
Differential proteomics analysis of ovarian tissue from *Takifugu bimaculatus* following TTX gavage: (**A**) Clustering heatmap; (**B**) Volcano plot; (**C**) KEGG enrichment analysis of DEPs; The red boxes: Key pathway of interest. (**D**) GO enrichment analysis of DEPs. DEPs: Differentially expressed proteins. Rad: Biological Process (BP); Purple: Cellular Component (CC); Orange: Molecular Function (MF). Full names of the GO terms are listed in Supplementary Table S6.

As shown in the clustering heatmap in Figure 7A, there were significant expression differences between the control and TTX groups in ovarian tissue, with good intra-group sample reproducibility. The volcano plot illustrates the distribution of DEPs between the two groups; a total of 179 significantly altered proteins were identified, of which 162 were upregulated and 17 were downregulated (Figure 7B). Subsequent enrichment analysis of the DEPs (Figure 7C,D) revealed that they are components of the cytoskeleton, vesicular membranes, and myosin complexes, and perform functions such as redox activity, adhesion, and structural molecule activity. KEGG enrichment further indicated that DEPs are primarily involved in extracellular matrix-receptor interactions and focal adhesion. These results suggest that the cell membrane and cytoskeleton play important roles in TTX transport and accumulation in pufferfish ovarian tissue.

### 2.4. Transcriptome-Proteome Correlation Analysis

**Figure 8:**
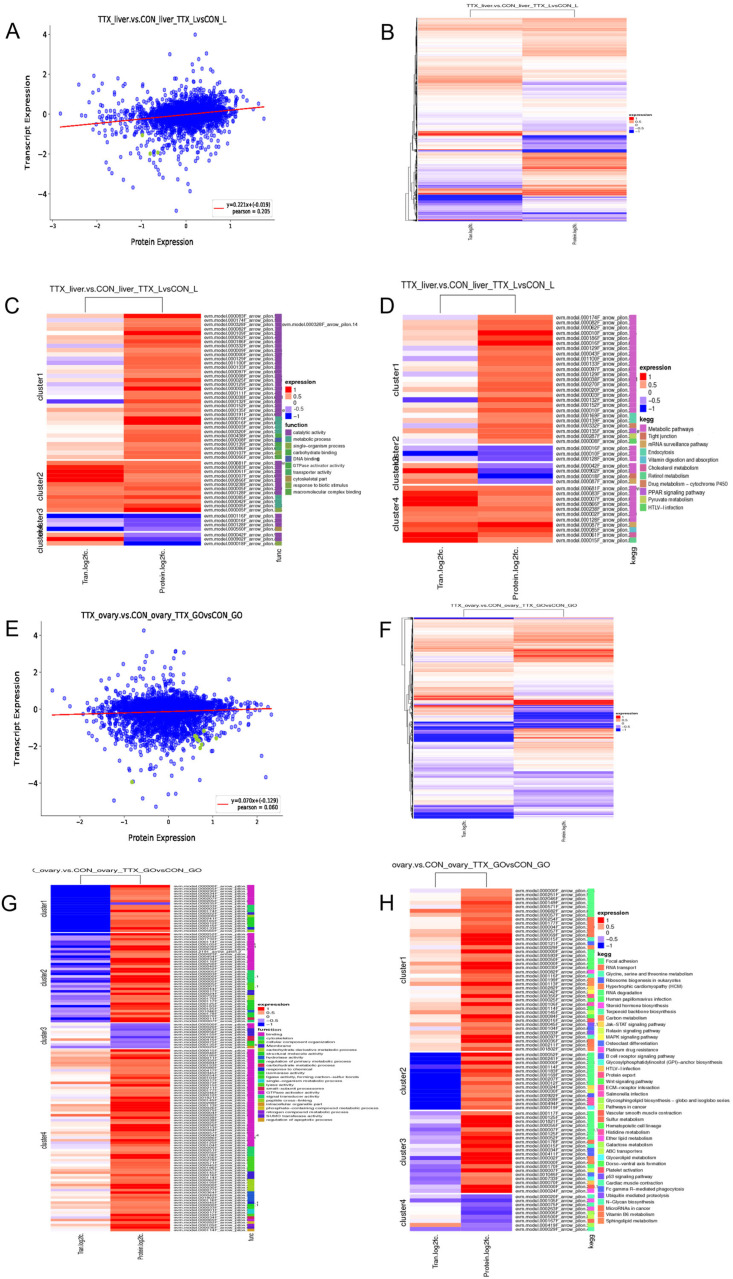
Integrated transcriptomic and proteomic analysis of liver and ovarian tissues from *Takifugu bimaculatus* following TTX gavage. Clusters: A collection of biological entities (such as genes, proteins, metabolites, etc.) that share similar expression patterns, functional characteristics, or regulatory relationships. (**A**) Correlation between DEGs and DEPs in liver tissue. Each point is representative of a protein; green points indicate proteins with significant differences in expression levels, while blue points indicate proteins with no significant differences in expression levels. The x-axis of the figure displays the fold change (log2 value) of the corresponding protein in the proteomics data, whilst the y-axis displays the fold change (log2 value) of the corresponding gene in the transcriptomics data; (**B**) Clustering heatmap of DEGs and DEPs in liver tissue; (**C**) GO enrichment analysis of DEGs and DEPs in liver tissue; (**D**) KEGG enrichment analysis of DEGs and DEPs in liver tissue. (**E**) Correlation between DEGs and DEPs in ovarian tissue; (**F**) Clustering heatmap of DEGs and DEPs in ovarian tissue; (**G**) GO enrichment analysis of DEGs and DEPs in ovarian tissue; (**H**) KEGG enrichment analysis of DEGs and DEPs in ovarian tissue. The original image can be found in Supplementary Figure S1.

To comprehensively analyze the molecular networks and interactions involved in TTX transport and accumulation, this study conducted a correlation analysis between the transcriptome and proteome (Figure 8). The results showed a weak positive correlation between the transcriptome and proteome in liver tissue, with a correlation coefficient of 0.205, while no correlation was observed between the transcriptome and proteome in ovarian tissue (Figure 8A,E). Clustering heatmaps also revealed significant expression differences between the two groups (Figure 8B,F). The DEGs and DEPs were grouped into four clusters and subjected to enrichment analysis, including KEGG pathway enrichment analysis and GO functional annotation analysis.

GO functional enrichment analysis (Figure 8C) revealed that DEGs/DEPs in liver tissue transcriptomes and proteomes were primarily enriched in metabolic processes, involved in cytoskeletal organization, carbohydrate binding, and macromolecular complex binding, and performed functions such as catalytic activity, GTP kinase activity, and transport activity. Clusters 1 and 4 exhibited opposite trends in differential expression, while Clusters 2 and 3 showed similar trends, with catalytic activity being the most significantly enriched. KEGG pathway enrichment analysis (Figure 8D) revealed that both groups of samples were jointly enriched in pathways related to metabolism, tight junctions, mRNA regulation, endocytosis, cholesterol/retinol/pyruvate metabolism, and PPAR signaling pathways. The trends in differential expression were opposite between Clusters 1 and 3, and identical between Clusters 2 and 4. Among these, the metabolic pathway showed the most significant enrichment.

GO functional enrichment analysis (Figure 8G) revealed that DEGs/DEPs in ovarian tissue transcriptomes and proteins were primarily enriched in various metabolic processes, as well as functions related to the cytoskeleton, organelle assembly, structural molecular activity, isomerase activity, SUMOylation, and adhesion. Among the clusters, only Clusters 4 and 5 showed similar trends in differential expression, while the others were opposite. Among these, the adhesion function was the most significantly enriched. KEGG pathway enrichment analysis (Figure 8H) revealed that both groups of samples were commonly enriched in cell adhesion, steroid hormone biosynthesis, terpenoid biosynthesis, ABC transporters, and the Wnt signaling pathway. Among these, cell adhesion was the most significantly enriched. In all four clusters, the expression trends between the two groups of samples were opposite. Taken together, these results suggest that the molecular mechanisms involved in TTX transport and accumulation in pufferfish are complex, and that there is a time lag between transcription and translation.

## 3. Discussion

The liver is a key organ for the transport and accumulation of TTX in pufferfish. Studies have shown that the form of TTX and the route of administration influence its transfer and accumulation in the liver. For instance, intramuscularly injected TTX crude extract (from toxic pufferfish ovaries) remained in the liver at higher concentrations for longer durations than the purified TTX standard [21]. Similarly, in feeding experiments with TTX-laced feed, pufferfish continued to retain TTX in their livers for an extended period even after ceasing to consume TTX-containing food [22]. Wang et al. [23] compared oral and intramuscular administration in hybrid pufferfish (*Takifugu rubripes* × *T. porphyreus*): oral administration resulted in hepatic TTX peaking at 1–24 h with gradual decline, whereas intramuscular injection produced an earlier peak at 8 h with shorter retention time. Notably, multiple studies have demonstrated that blood serves as a critical transport medium for TTX [24,25]. Melnikova et al. [26] demonstrated that TTX absorbed by the gastrointestinal tract is first transported to the liver via the bloodstream and then rapidly enters other tissues and organs. Consistent with these findings, our oral administration experiments revealed comparable TTX dynamics. Initially, TTX was primarily distributed in the gastrointestinal tract and bloodstream; over time, it transferred to tissues, and as this process progressed, TTX levels in the liver began to rise, reaching a peak at 12 h after administration.

The ovary is one of the primary tissues for the specific accumulation of TTX in pufferfish, with its toxicity peaking during the reproductive season. Specifically, Itoi et al. [27] analyzed the seasonal variations in TTX levels in the pufferfish *Takifugu niphobles*, showing that both males and females exhibited significantly higher TTX levels during the maturation (4 month) and spawning (5–7 months) periods compared to other times of the year. Beyond seasonal fluctuations, studies have reported that certain pufferfish, such as *Takifugu niphobles*, accumulate TTX in their ovaries and release it as a sex pheromone, which attracts male *Takifugu niphobles* to spawning grounds for mating, thereby increasing the chances of fertilization [11,28]. Furthermore, all TTX in the fertilized eggs of *Takifugu bimaculatus* originates from the female parent’s eggs, helping pufferfish embryos and larvae defend against external predators; this is presumed to be one of the key reasons for the massive accumulation of TTX in pufferfish ovaries [27,29]. In our observations, liver TTX levels decreased at Day 7, coinciding with peak TTX accumulation in the ovaries. This is consistent with previous reports indicating that TTX in pufferfish is primarily concentrated in the liver, ovaries, and skin tissues [30,31].

The skin is also a major site for TTX accumulation. As early as 1986, Kodama et al. [32] identified specific exocrine glands or gland-like structures in the skin of several *Takifugu* species (*T. poecilonotus*, *T. niphobles*, and *T. vermiculare radiatum*) and detected high concentrations of TTX within these glands. The skin is the primary organ through which pufferfish interact with the external environment, and the accumulation of TTX aligns with the defensive characteristics of the pufferfish’ s body surface [31]. It has been reported that some potential predatory fish immediately spit out newly hatched pufferfish larvae upon encountering TTX in their skin [33]. Furthermore, a previous study confirmed that excess TTX in the liver is transferred to the epidermis in juveniles tiger pufferfish [25]. The trends observed in our study align with these previous reports. Specifically, TTX levels in the liver decreased after 12 h; whereas cutaneous TTX continued to accumulate, peaking at day 7.

The weak correlation between transcriptomic and proteomic data observed in this study has also been widely reported in previous research [34,35]. Several factors may account for this discrepancy. Olivares-Hernández et al. [36] found that differences in codon composition play a key role. Ponomarenko et al. [37] proposed that the rate at which ribosomes move along mRNA, the number of free ribosomes in the cell, tRNA availability, transcript localization, secondary structure and the technical characteristics of experimental methods all significantly influence the levels of corresponding gene transcripts and proteins in comparative analyses. Furthermore, studies have shown that proteins have a more complex chemical structure than nucleic acids composed of four nucleotides linked by identical phosphodiester bonds, which endow them with a broader range of functions. At the same time, metabolic pathways in living organisms exhibit branching and cyclic patterns, with most reactions being non-linear, which may exacerbate expression discrepancies between the transcriptome and the proteome [38]. Despite this quantitative discordance at the individual gene level, both datasets converged on shared functional themes.

At the functional pathway level in the liver, multi-omics data collectively point to the central role of amino acid metabolism and transmembrane transport. Proteomics analysis revealed significant enrichment in cysteine and methionine metabolism, while transcriptomics analysis showed upregulation of glutathione and phenylalanine metabolic pathways. Cysteine is the rate-limiting precursor for glutathione (GSH) synthesis. As the most significant intracellular antioxidant, the enhanced metabolism of GSH suggests that the liver may counteract TTX-induced oxidative stress by increasing its antioxidant capacity [39]. In addition, the methionine cycle is involved in methyl donor metabolism [40] and may be related to the biotransformation of toxins or the excretion of detoxification products. Given that TTX is a positively charged alkaloid, its transmembrane transport may depend on ion gradients or specific carrier proteins; this view is further supported by the isolation of a binding protein (PSTBP) involved in TTX transport from the plasma of *Takifugu pardalis* by Yotsu-Yamashita et al. [41,42]. The GO analysis of the transcriptome in this study revealed that upregulated genes were significantly enriched in sulfide/sodium ion/dicarboxylic acid transport and sodium-dicarboxylic acid transporter activity. Sodium-dependent transporters (such as sodium-dicarboxylic acid cotransporters) may be involved in the TTX transport process either indirectly or directly. Concurrently, upregulation of the renin-angiotensin system may influence TTX accumulation and transport rates by regulating hepatic blood flow.

In addition, the anabolic metabolism of various carbohydrates in the liver was significantly upregulated, indicating that the transport and accumulation of TTX consume energy either directly or indirectly. Steroid biosynthesis and the terpene backbone biosynthesis pathway were significantly downregulated; since the terpene backbone pathway is upstream of the steroid biosynthesis pathway, this directly affects steroid biosynthesis. Cholesterol abundance influences the structure of lipid rafts on the membrane, which serve as anchoring sites for many transport proteins (such as P-glycoprotein and multidrug resistance-associated protein) [43]. Lu et al. [44] detected the presence of TTX in the gallbladder of *Takifugu obscurus*. Therefore, fluctuations in steroid metabolism may affect the liver’s efficiency in accumulating or transporting TTX and its derivatives to the gallbladder and ovaries.

Unlike the liver, the ovary exhibited a strong capacity for active accumulation throughout the 168 h period of TTX exposure. TTX affects multiple metabolic processes (nucleotidic acid metabolism, phosphate metabolism, and N-acetylglucosamine metabolism), involving the anchoring of the cytoskeleton to the plasma membrane and glycolysis. Furthermore, KEGG enrichment analysis of the ovarian proteome significantly pointed to extracellular matrix-receptor interactions and focal adhesion pathways, while GO analysis also highlighted cortical actin cytoskeleton and endosomal vesicle formation. Ovarian cells may promote intercellular transport and regulation by reorganizing cytoskeletal networks, enhancing intercellular adhesion junctions, and activating endocytosis [45,46]. Rearrangement of the cytoskeleton might facilitate the directed transport and storage of TTX-containing vesicles within the cell, which hypothetically prevent the toxin from interfering with the replication of genetic material in the nucleus. This offers a possible explanation for the observed downregulation of genes associated with meiosis and homologous chromosome segregation. Concurrently, alterations in nicotinamide, phosphate, and N-acetylglucosamine metabolism may signify metabolic adaptations within the ovary, presumably aimed at satisfying the energy and substrate requirements for intensive vesicular transport and membrane remodeling.

**Figure 9:**
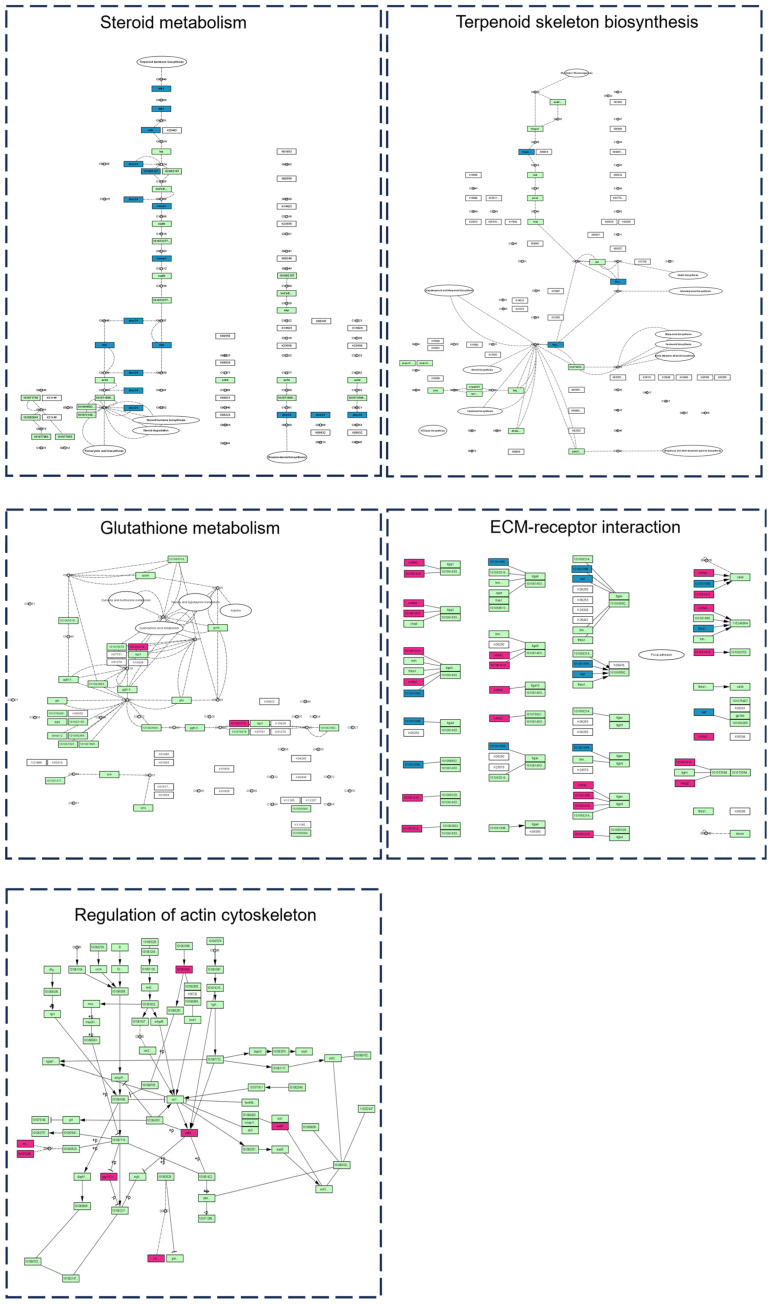
Key signaling pathways involved in the regulation of DEGs and DEPs in the liver and ovary tissues of *Takifugu bimaculatus* following TTX gavage. Red: upregulated; Blue: downregulated. Green: The gene or protein is present in *Takifugu* species; White: The presence of the gene or protein in *Takifugu* species remains unconfirmed. The original image can be found in Supplementary Figure S2.

The downregulation of immune response and meiosis-related pathways among DEGs may indicate that, during periods of high toxin accumulation, the ovaries potentially suppress certain immune responses to avoid possible toxin rejection, while concomitantly slowing down cell division to prioritize TTX storage. Furthermore, phototransduction is significantly enriched in non-visual tissues. Previous reports have indicated the expression of visual protein genes such as opn4 and opn5 in non-visual tissues of fish [47]; these genes may promote TTX-associated vesicular transport by regulating intracellular calcium ion concentrations. Additionally, TTX transporters are also present in the ovaries of pufferfish. Yin et al. [48] identified a tetrodotoxin-binding protein in the ovaries of *Takifugu pardalis*; this protein is a domain of the vitellogenin VWD and possesses tetrodotoxin-binding activity. Concurrently, our laboratory’s previous work has already provided a detailed characterization of the amino acids involved in the binding of TTX to VWD in the ovaries of *Takifugu xanthopterus* [49]. In this study, transcriptomic and proteomic correlation analyses jointly identified ABC transporters as enriched targets. Zhang et al. [50] also found that ABC family mRNAs were differentially expressed in the livers of *Takifugu rubripes* following TTX enrichment. These findings suggest that ABC transporters may be involved in the transport and accumulation of TTX within pufferfish. Figure 9 summarizes the key regulatory pathways of DEGs and DEPs in the liver and ovarian tissues of *Takifugu bimaculatus* following TTX gavage.

## 4. Materials and Methods

### 4.1. Experimental Animals and Husbandry Conditions

Two-year-old, sexually mature female *Takifugu bimaculatus* (body length: 22 ± 2 cm; body weight: 300 ± 30 g) were obtained from the Zhangpu Pufferfish Farming Base (Zhangpu, China). Fish were acclimated in 100 L fiberglass tanks (six individuals per tank) supplied with aerated seawater (salinity: 30–32 ppt; temperature: 22 ± 1 °C; dissolved oxygen: >6 mg/L) under a 12 h light/12 h dark photoperiod for 7 days prior to experimental manipulation.

### 4.2. TTX Administration and Tissue Sampling

Following the acclimation period, fish were randomly assigned to experimental (TTX-treated) or control (vehicle-treated) groups. Tetrodotoxin crude extract was initially dissolved in sodium acetate buffer (pH 4.0) to prepare a stock solution (25 mg/mL), which was subsequently diluted with sterile phosphate-buffered saline (PBS, pH 7.4) to a working concentration of 600 μg/mL. Fish were anesthetized by immersion in 0.01% (*v*/*v*) clove oil for 5–10 min until opercular movement ceased. A flexible gavage tube (outer diameter: 1.2 mm) was attached to a 1 mL sterile syringe, and the working solution was administered via oral gavage (0.5 mL per fish, equivalent to a nominal dose of 1 μg TTX/g body weight). Control fish received an equivalent volume of sterile, endotoxin-free PBS. Immediately following administration, fish were transferred to clean, aerated seawater for recovery and monitored until resumption of normal swimming behavior.

The experimental design incorporated seven temporal sampling points (0, 3, 6, and 12 h; 1, 2, and 7 days post-administration; *n* = 4 per group per time point). At each designated time point, fish were euthanized by immersion in anesthetic overdose (0.05% clove oil) followed by cervical transection. Body morphometrics (total length and weight) were recorded, and external surfaces were rinsed with sterile deionized water and blotted dry. Tissues were collected in the following sequence to minimize cross-contamination: blood (via caudal venipuncture), eyes, skin, muscle, kidneys, stomach, intestine, ovaries, and liver. Blood samples were collected using heparinized syringes and centrifuged (3000× *g*, 10 min, 4 °C) to obtain plasma. Solid tissues were immediately snap-frozen in liquid nitrogen and stored at −80 °C until subsequent analysis. All samples were allocated unique identifiers and processed within 30 min of euthanasia to ensure analyte stability.

### 4.3. Quantification of TTX Content

Tetrodotoxin was extracted from pufferfish tissues according to the National Food Safety Standard of China (GB 5009.206-2016: Determination of Tetrodotoxin in Aquatic Products) [18]. Quantitative analysis of TTX was performed using the enzyme-linked immunosorbent assay (ELISA) method. The antigen (BSA-TTX) was coated at 4 °C overnight, blocked, air-dried, and stored at −20 °C. Standards and labeled antibodies were added, and the plate was incubated at 25 °C for 30 min. After washing the plate, TMB was added for color development, followed by stopping with sulfuric acid. The absorbance was measured at 450 nm. The tetrodotoxin concentration in the samples was quantified using a standard curve.

**Figure 10:**
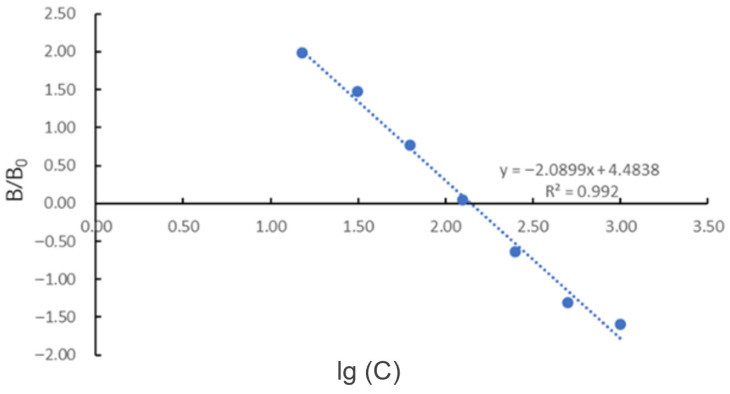
Standard curve of TTX by ELISA.

A standard curve was plotted with the logarithm (base 10) of the concentration of the tetrodotoxin standard series working solutions as the *x*-axis, and the ratio of the absorbance value (B) of the tetrodotoxin standard series working solutions to the absorbance value (B0) of the 0 μg/L tetrodotoxin standard working solution as the *y*-axis. The standard curve for the TTX standard samples was obtained as y = −2.0899x + 4.4838, with an R^2^ value of 0.992 (Figure 10), satisfying the experimental requirements for the ELISA.

### 4.4. Transcriptomic Analysis

Transcriptome sequencing was performed on liver tissue 24 h after oral administration of TTX or PBS, and on ovarian tissue 168 h after administration, with three replicate samples per group.

#### 4.4.1. RNA Extraction and Sequencing

Total RNA was extracted from tissue samples using TRIzol reagent (Invitrogen, Carlsbad, CA, USA) according to the manufacturer’s protocol. RNA purity (A260/A280: 1.8–2.1; A260/A230 > 2.0) was assessed using a NanoDrop spectrophotometer (Thermo Fisher Scientific, Waltham, MA, USA), and integrity (RIN ≥ 7.0) was verified using an Agilent 2100 Bioanalyzer (Agilent Technologies, Palo Alto, CA, USA). Strand-specific cDNA libraries were constructed using the TruSeq Stranded mRNA Library Prep Kit (Illumina, San Diego, CA, USA) and sequenced on the NovaSeq 6000 platform (Illumina, San Diego, CA, USA) (150 bp paired-end reads, ≥6 Gb per sample).

#### 4.4.2. Transcriptome Data Analysis

Raw reads were processed with Trimmomatic (v0.39) to remove adapters and low-quality bases, and the resulting clean reads were aligned to the *Takifugu bimaculatus* genome (fTakRub1.2) using HISAT2 (v2.2.1). Gene-level counts were generated with featureCounts (v2.0.1). Principal component analysis (PCA) and hierarchical clustering heatmaps were performed to evaluate sample distribution and group relationships. Differential expression analysis was performed using DESeq2 (v1.34.0) with IHW correction. Genes with FDR < 0.05 and |log_2_FC| ≥ 2 were defined as DEGs. Functional enrichment was analyzed using clusterProfiler (v4.2.2) for GO terms and KEGG pathways (adjusted *p* < 0.05).

### 4.5. Proteomic Analysis

#### 4.5.1. Protein Extraction and Digestion

Proteins were extracted by acetone precipitation (−20 °C, overnight) and quantified using the BCA Protein Assay Kit (Thermo Fisher Scientific, Waltham, MA, USA). Protein samples (100 μg) were reduced with 10 mM dithiothreitol (DTT, 56 °C, 30 min), alkylated with 55 mM iodoacetamide (IAM, room temperature, 20 min, dark), and subsequently digested with trypsin (Promega, Madison, WI, USA) at a 1:50 enzyme-to-substrate ratio (37 °C, 16 h). The resulting peptides were desalted using C18 spin columns and lyophilized for LC-MS/MS analysis.

#### 4.5.2. LC-MS/MS Analysis

Peptide samples were resuspended in 0.1% formic acid and separated using a nanoflow UHPLC system (EASY-nLC 1200, Thermo Fisher Scientific, Waltham, MA, USA) coupled to a Q Exactive HF-X mass spectrometer (Thermo Fisher Scientific, Waltham, MA, USA). Chromatographic separation was performed on a reversed-phase C18 analytical column (15 cm × 75 μm, 3 μm particle size) with a 60 min linear gradient (4–28% acetonitrile in 0.1% formic acid). The mass spectrometer was operated in data-dependent acquisition (DDA) mode: full MS scans were acquired at 60,000 resolution (*m*/*z* 200–1800), followed by HCD fragmentation of the top 20 most intense precursor ions (resolution 15,000, normalized collision energy 28%, dynamic exclusion 30 s).

#### 4.5.3. Proteomics Data Analysis

Raw MS data were analyzed using MaxQuant (v2.0.1.0) against the *Takifugu bimaculatus* UniProt database. Carbamidomethylation of cysteine was set as a fixed modification, while methionine oxidation and N-terminal acetylation were set as variable modifications. The false discovery rate (FDR) was controlled at <1% at both peptide and protein levels (decoy database approach). Label-free quantification (LFQ) was performed using the LFQ algorithm, and differentially expressed proteins (DEPs) were identified based on |log_2_ fold change| > 0.58 (equivalent to 1.5-fold change) and adjusted *p* < 0.05 (Student’s *t*-test with Benjamini–Hochberg correction). Functional enrichment analysis (GO and KEGG) was subsequently conducted using the clusterProfiler package.

### 4.6. Molecular Validation

Gene expression was validated via RT-qPCR to confirm the RNA-seq analysis results. The EF1-α primer (GenBank accession number: MT160192) was used to amplify the internal reference gene, and qPCR data were calculated using the 2^−ΔΔCT^ method; the primer sequences are shown in Table 1.

## 5. Conclusions

TTX exhibits dynamic distribution and specific accumulation within the body of *Takifugu bimaculatus*; the liver, ovaries, and skin are the primary tissues in which it accumulates. Furthermore, complex regulatory mechanisms govern the transport and accumulation of TTX within the pufferfish. While the liver utilizes ion concentrations, lipid raft structures, and carrier proteins to accumulate TTX, it also possesses corresponding efflux (transport to the ovaries and skin) and metabolic mechanisms that control the TTX load within the tissue and maintain the body’s homeostasis. The ovaries, on the other hand, establish long-term storage reservoirs for TTX by remodeling the cytoskeleton, enhancing interactions with the extracellular matrix, and activating endocytic pathways. The weak correlation between the transcriptome and proteome reveals a significant time lag in transcriptional regulation during this process. This study not only elucidates the potential molecular mechanisms underlying tetrodotoxin accumulation but also provides a fundamental theoretical basis and data support for screening key toxin transporter genes, elucidating the metabolic mechanisms of TTX. Future research will focus on functional validation of key candidate genes (such as sodium-dicarboxylate transporters and cytoskeletal regulators) and employ multi-time point dynamic monitoring to comprehensively map the spatiotemporal profile of TTX transport.

## Figures and Tables

**Table 1:** Primers sequence.

Genes	Forward Primer	Reverse Primer
MMP2	AAGATTGGCTCGGCTGTTGA	ATCAGCCTGTGTGTCTGCTC
COL17A1	TTACGGCGTTCCGAAGAACA	TCAGTGTTTCAGTCTCCCGC
COLGALT1	TCACCAAAGGGGAGCTAGGA	TAAGTCCATCAAACGGCGCT
CYP51A1	AGAGGCGGCACGAGTGAAG	ATCCCTGGAAAGACACATTAGTTGAG
DHCR7	TTTCCGCTGGTTTTCTCCCA	GACAGGTTGATGAGCGTCCA
DHCR24	TGCTGCCTCTTTCCGTCCTC	TTACTACCATCCTTCCTCCACTCAC
ERG28	AGAAACTTTACACAGGCACACCAG	GCACAGCGGATGATAGATGACAG
Novel03337	AGAGCAGAGCCAGTTCCAGTG	ACCAGCAGCAGGATGAAGAGG
FDFT1	GTTTTGCAGCGGTGATCCAG	CATCGGGACCTTCGTGTTCA
HMGCR	TGCTGACTCGTCTCTTCCGTATG	GGTAAGCGTGACTGTGCCAAC
HMGCS1	AGCACGCATACGACTTCTACAAAC	GGTCCAGAGCACTCAGGTAGC
IDI1	AACACGGAACACTTGGATGAGAAG	ATGTTGGAGTTAAGGTGGCAGTTC
LSS	GCCCATAAGTTCCTCACCATCAC	CACCCTTGTTCATCTGCCTGTAG
RPS3	TGCGTAGGGCGTGCTATGG	CGAACTTCATGGACTTGGCTCTC
MSMO1	CTGCTGAAGACAGACAGCCA	GCGCTTCATTCGCAGTAAGG
COL14A1	CTCACCGCCCGAAGATACTC	AAGTTTTGCGCCGTCACTTC
FN1	TAAACCCGATGTCCCCAAGC	GATCCTGTAGCCGGTGATGG
TIMP2	GGGAACCCCGTCAAACAGAT	GATGAAGTCGCACAGGGTGA
TFPI	GACCATCAGGTGCAGACGAA	TTGTACGTGAACCTCCGCTC
EF1-*α*	ACTGAGGTGAAGTCTGTGGAAATGC	TTTGGTGGGTCGTTCTTGCTGTC

## Data Availability

The original data presented in the study are openly available in NCBI at PRJNA1453776.

## Supplementary Materials

The following supporting information can be downloaded at: https://www.mdpi.com/article/10.3390/md24050172/s1, Table S1: Quality of liver data output, Table S2: Quality of ovary data output; Table S3–S6: The full name of GO term; Supplementary Figure S1/S2: Figure 8 and Figure 9 original image.
