# Supplementary material for: Multi-Omics Reveals Tetrodotoxin Transport and Accumulation Mechanisms in Takifugu bimaculatus
Source: Mar Drugs. 2026 May 10;24(5):172. doi: 10.3390/md24050172 (PMC13208381; doi:10.3390/md24050172)
Supplement: Supplementary file 1 [file marinedrugs-24-00172-s001.zip › marinedrugs-4281737-Supplementary Tables S1 and S2.pdf]

Table S1 Quality of liver data output

Sample name	Raw reads	Clean reads	Clean bases	Error rate(%)	Q20 (%)	Q30 (%)	GC content (%)
CON_L1	63290566	62426378	9.36G	0.03	97.79	93.92	51.12
CON_L2	55997282	54743818	8.21G	0.03	97.51	93.34	51.47
CON_L3	68517516	65354870	9.8G	0.03	97.72	93.79	51.67
TTX_L1	56441700	54236176	8.14G	0.03	97.80	93.98	51.29
TTX_L2	59721132	58445772	8.77G	0.03	97.69	93.72	51.93
TTX_L3	62273790	59946676	8.99G	0.03	97.64	93.51	51.71

Table S2 Quality of ovary data output

Sample name	Raw reads	Clean reads	Clean bases	Error rate(%)	Q20 (%)	Q30 (%)	GC content (%)
CON_O1	65385714	64114462	9.62G	0.03	97.52	93.47	52.79
CON_O2	67875982	66735148	10.01G	0.03	97.31	92.96	52.27
CON_O3	65305350	64188398	9.63G	0.03	97.57	93.53	52.33
TTX_O1	47666308	46868678	7.03G	0.03	97.63	93.60	52.16
TTX_O2	59388174	58175494	8.73G	0.03	97.50	93.40	52.46
TTX_O3	55316466	54200352	8.13G	0.03	97.54	93.48	52.20
