# Supplementary figures and images for: Multi-Omics Reveals Tetrodotoxin Transport and Accumulation Mechanisms in Takifugu bimaculatus
Source: Mar Drugs. 2026 May 10;24(5):172. doi: 10.3390/md24050172 (PMC13208381; doi:10.3390/md24050172)

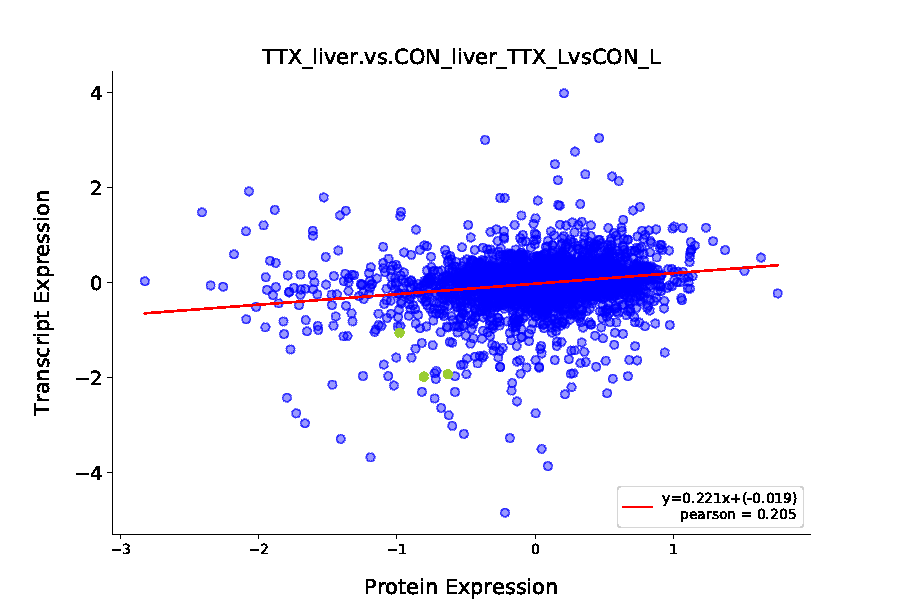

Supplement: Supplementary file 1 [file marinedrugs-24-00172-s001.zip › Supplementary figure/Figure S1/Figure S1A.png]

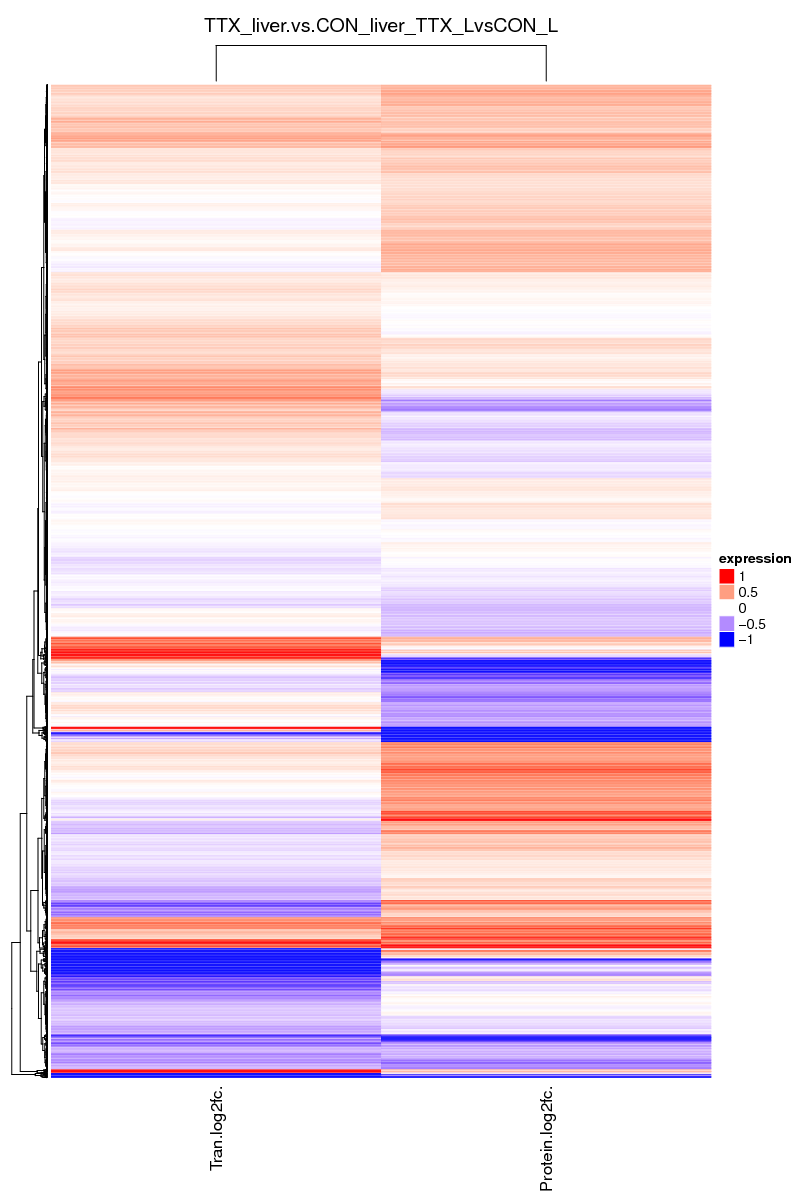

Supplement: Supplementary file 1 [file marinedrugs-24-00172-s001.zip › Supplementary figure/Figure S1/Figure S1B.png]

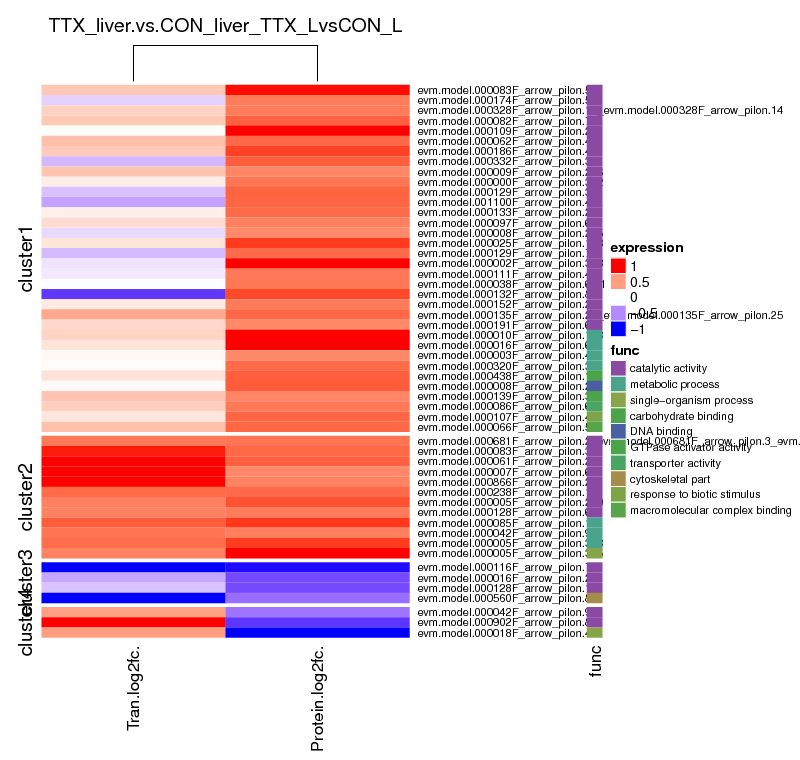

Supplement: Supplementary file 1 [file marinedrugs-24-00172-s001.zip › Supplementary figure/Figure S1/Figure S1C.png]

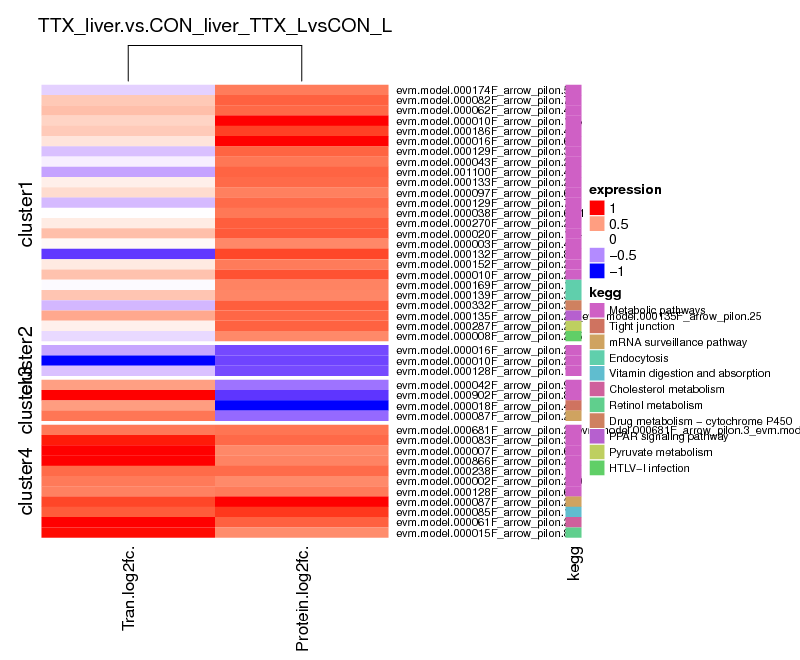

Supplement: Supplementary file 1 [file marinedrugs-24-00172-s001.zip › Supplementary figure/Figure S1/Figure S1D.png]

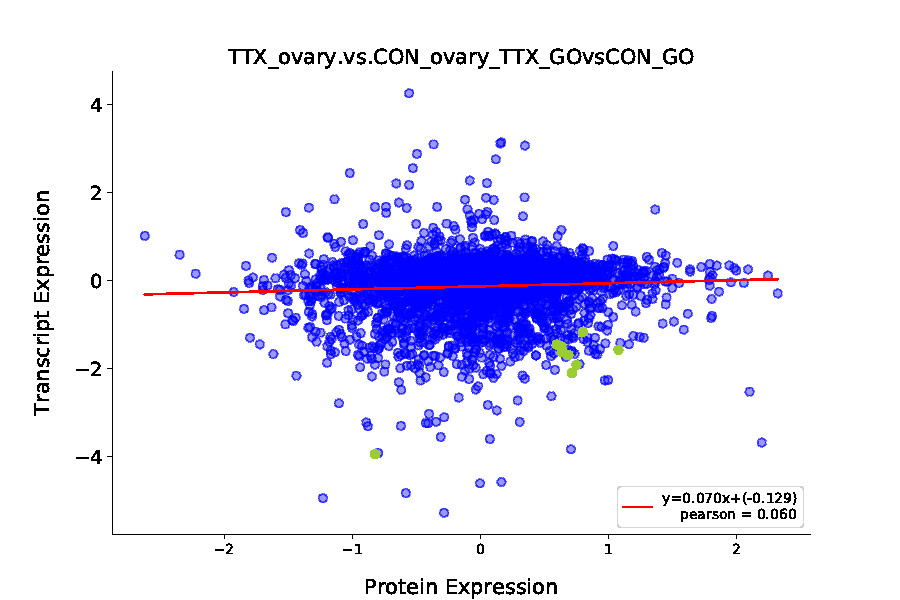

Supplement: Supplementary file 1 [file marinedrugs-24-00172-s001.zip › Supplementary figure/Figure S1/Figure S1E.png]

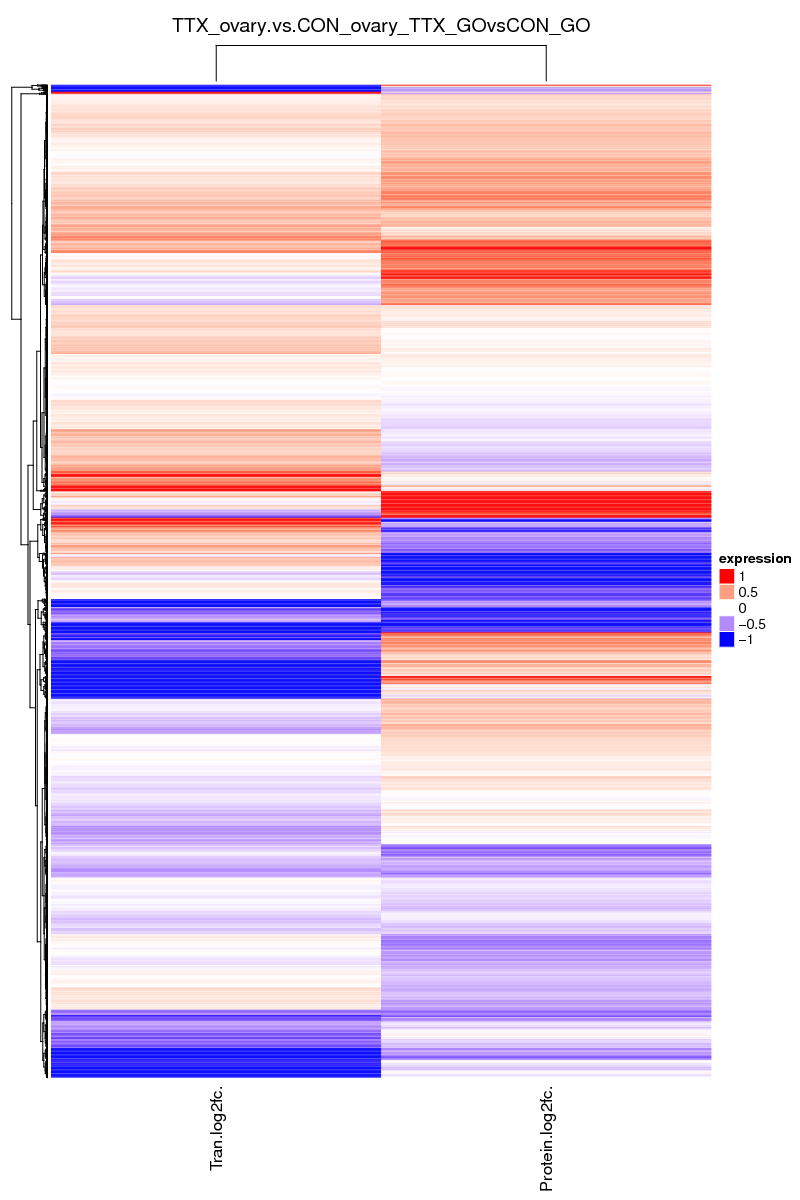

Supplement: Supplementary file 1 [file marinedrugs-24-00172-s001.zip › Supplementary figure/Figure S1/Figure S1F.png]

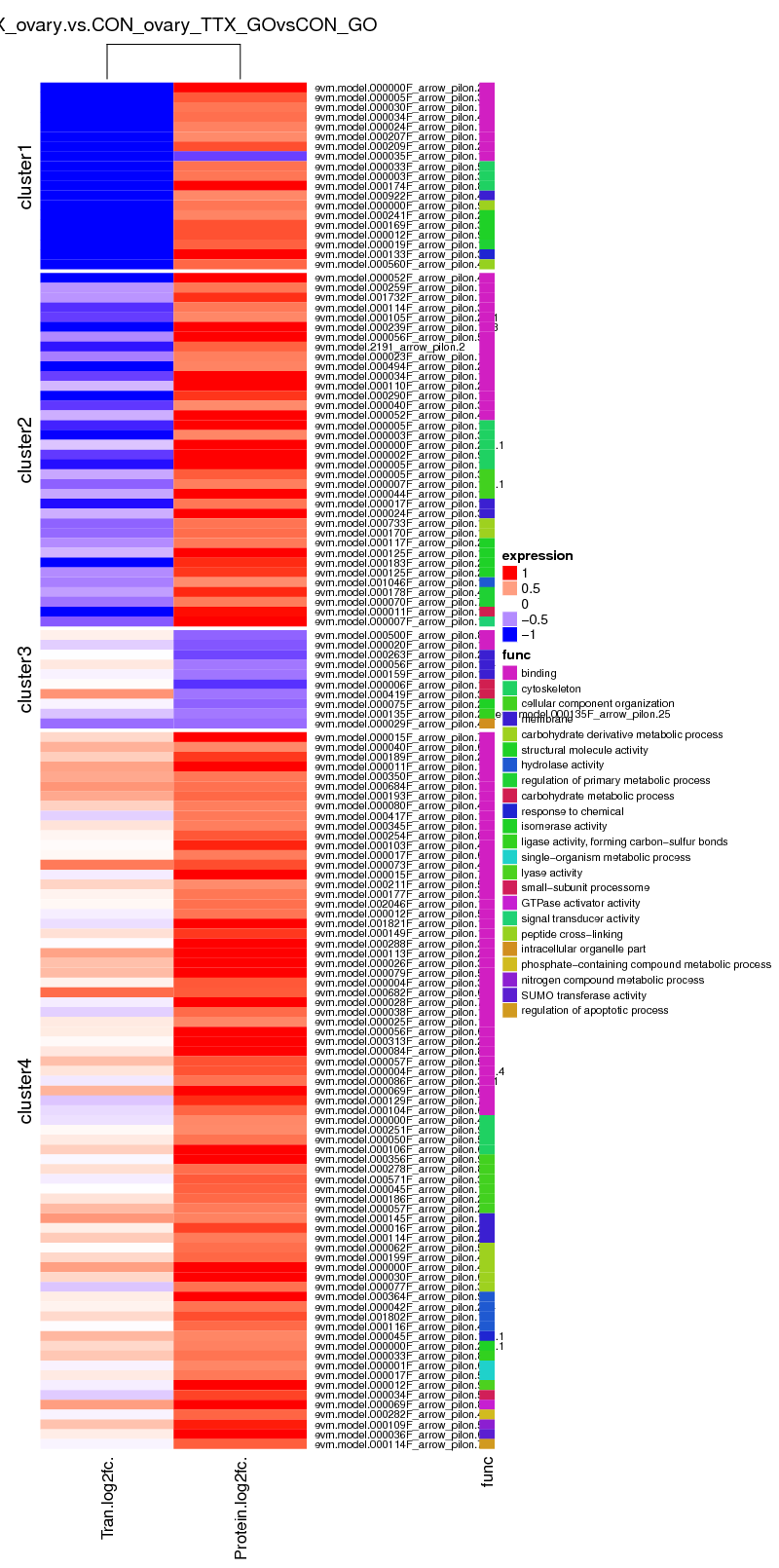

Supplement: Supplementary file 1 [file marinedrugs-24-00172-s001.zip › Supplementary figure/Figure S1/Figure S1G.png]

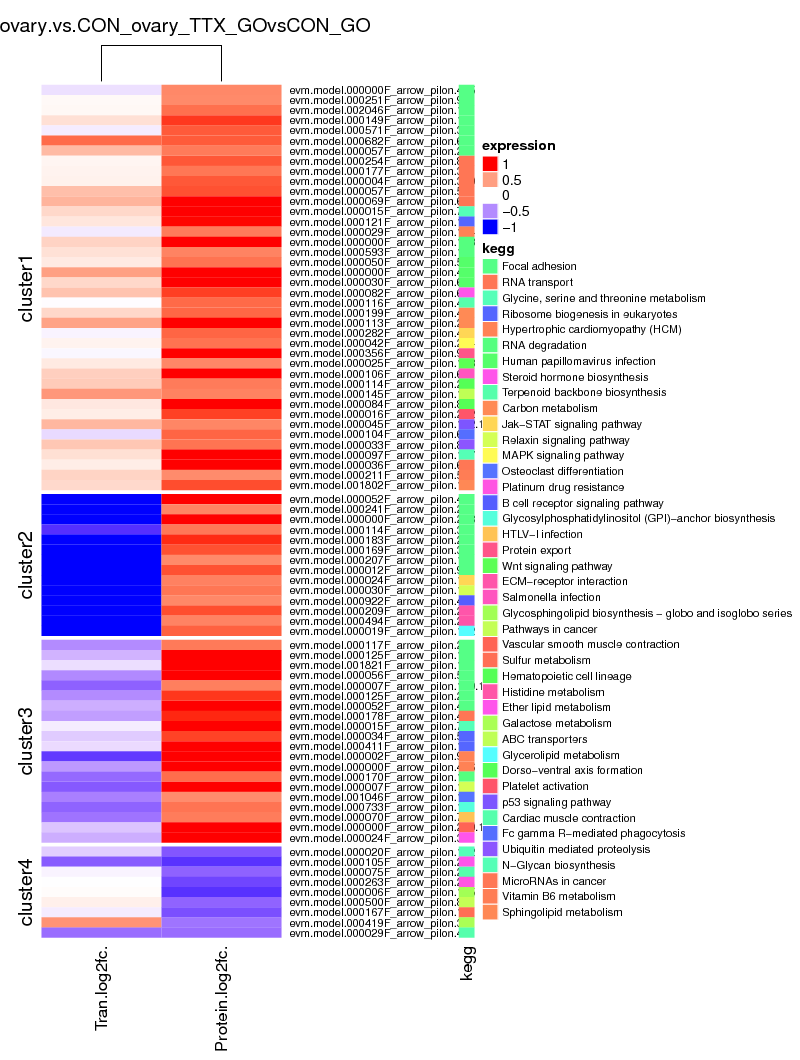

Supplement: Supplementary file 1 [file marinedrugs-24-00172-s001.zip › Supplementary figure/Figure S1/Figure S1H.png]

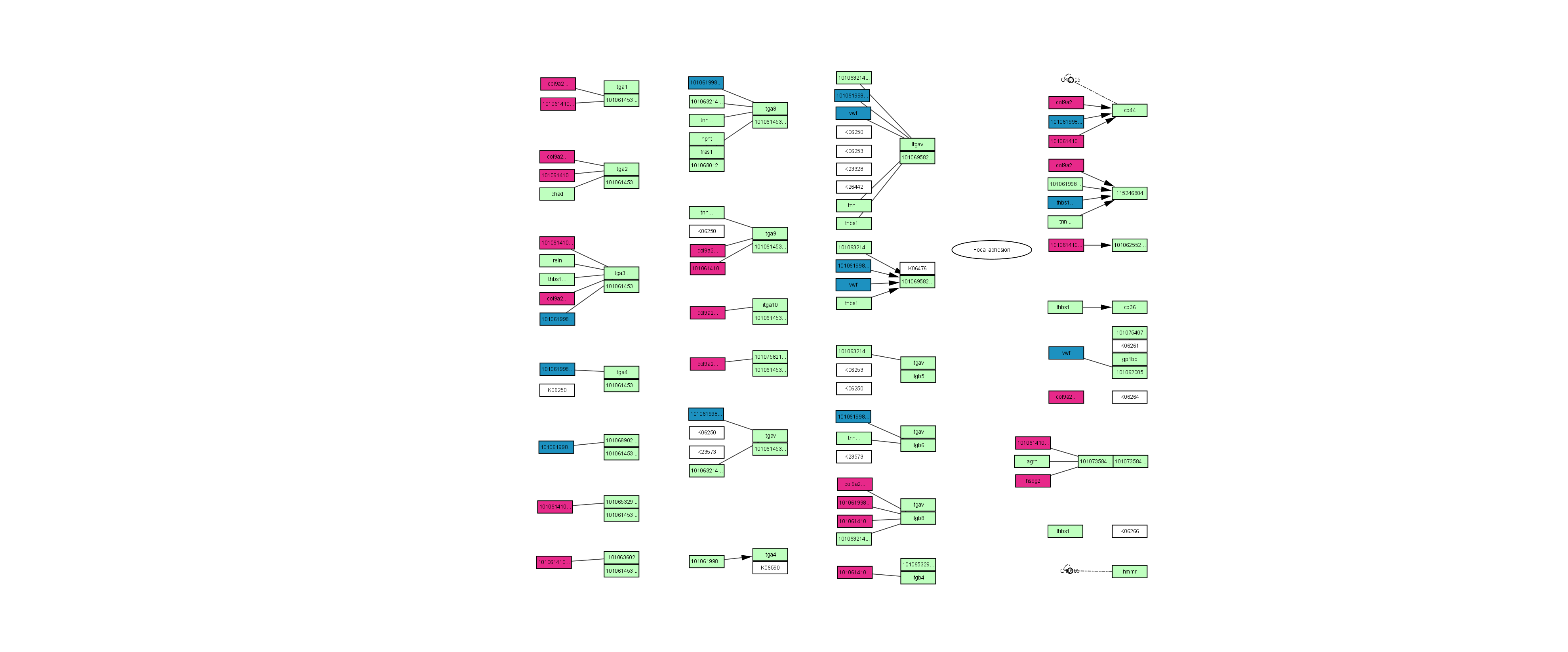

Supplement: Supplementary file 1 [file marinedrugs-24-00172-s001.zip › Supplementary figure/Figure S2/ECM-receptor interaction.png]

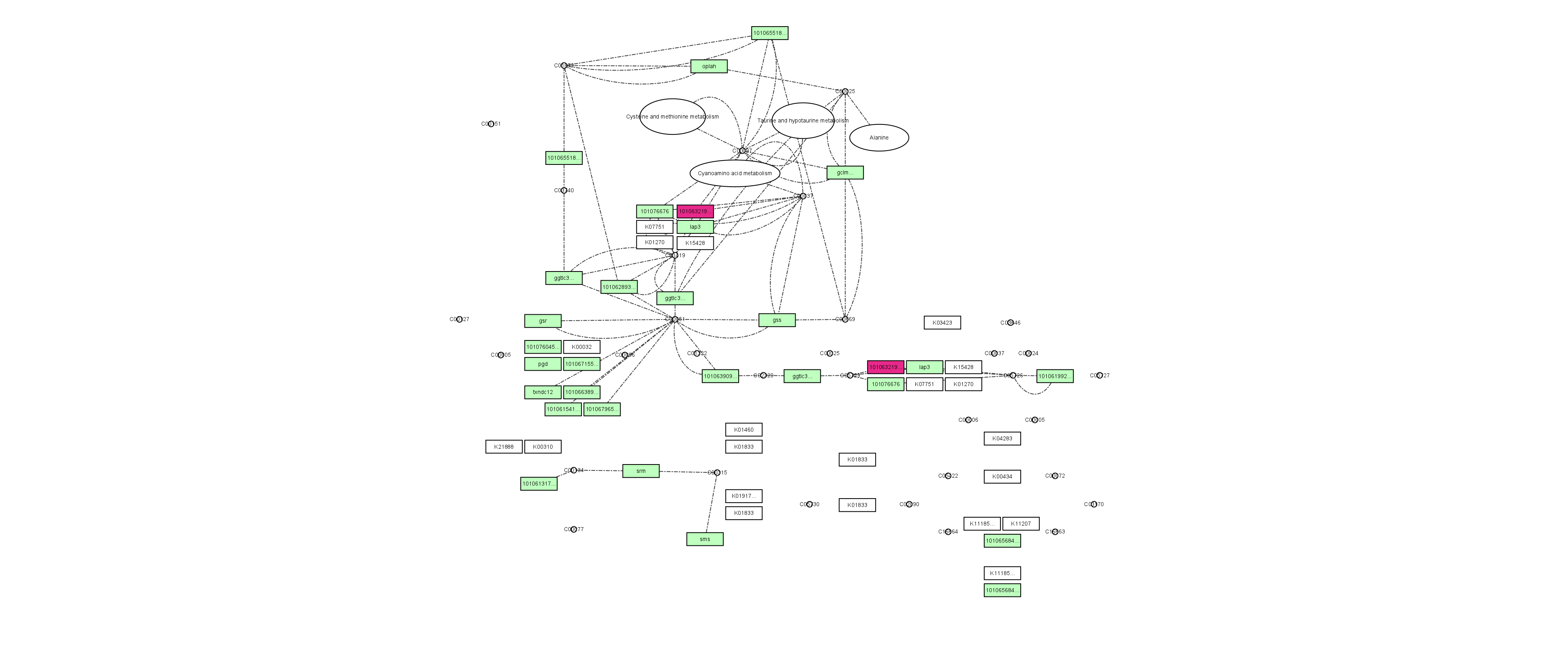

Supplement: Supplementary file 1 [file marinedrugs-24-00172-s001.zip › Supplementary figure/Figure S2/Glutathione metabolism.png]

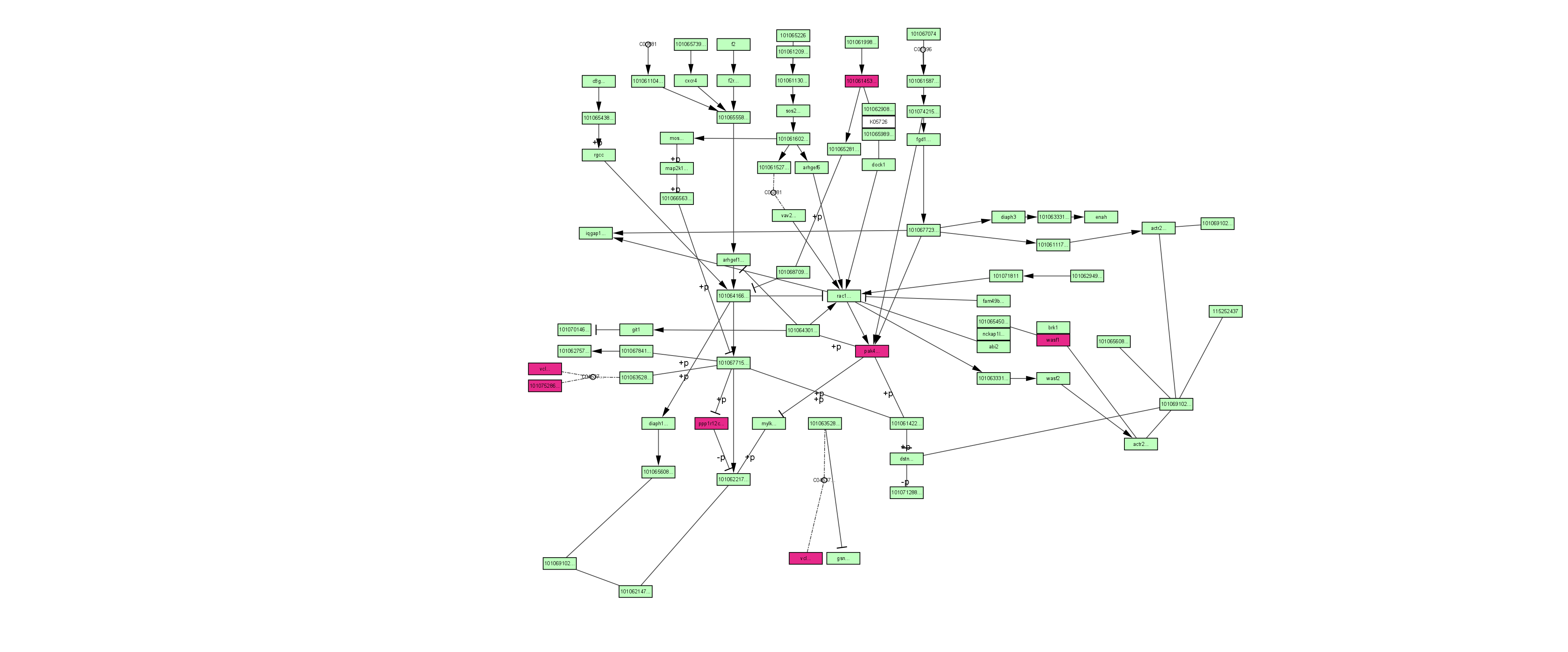

Supplement: Supplementary file 1 [file marinedrugs-24-00172-s001.zip › Supplementary figure/Figure S2/Regulation of actin cytoskeleton.png]

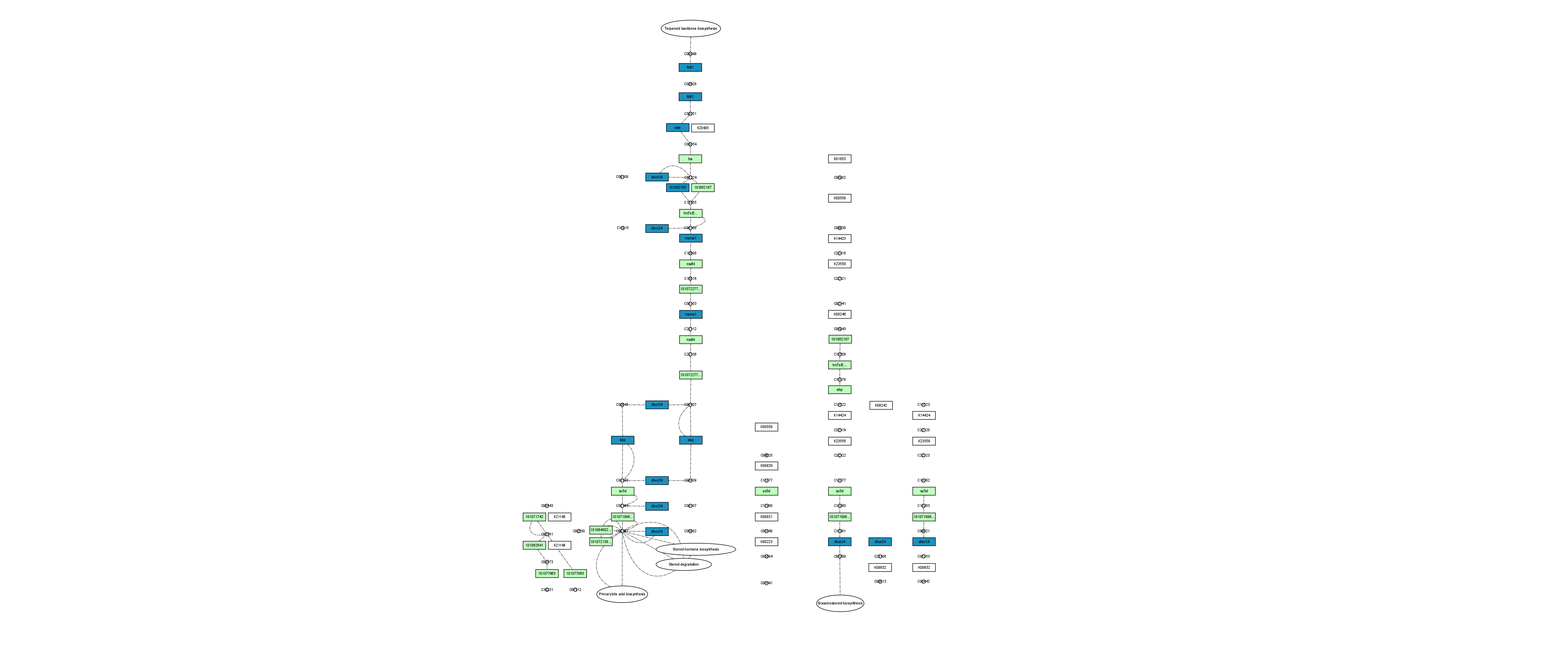

Supplement: Supplementary file 1 [file marinedrugs-24-00172-s001.zip › Supplementary figure/Figure S2/Steroid metabolism.png]

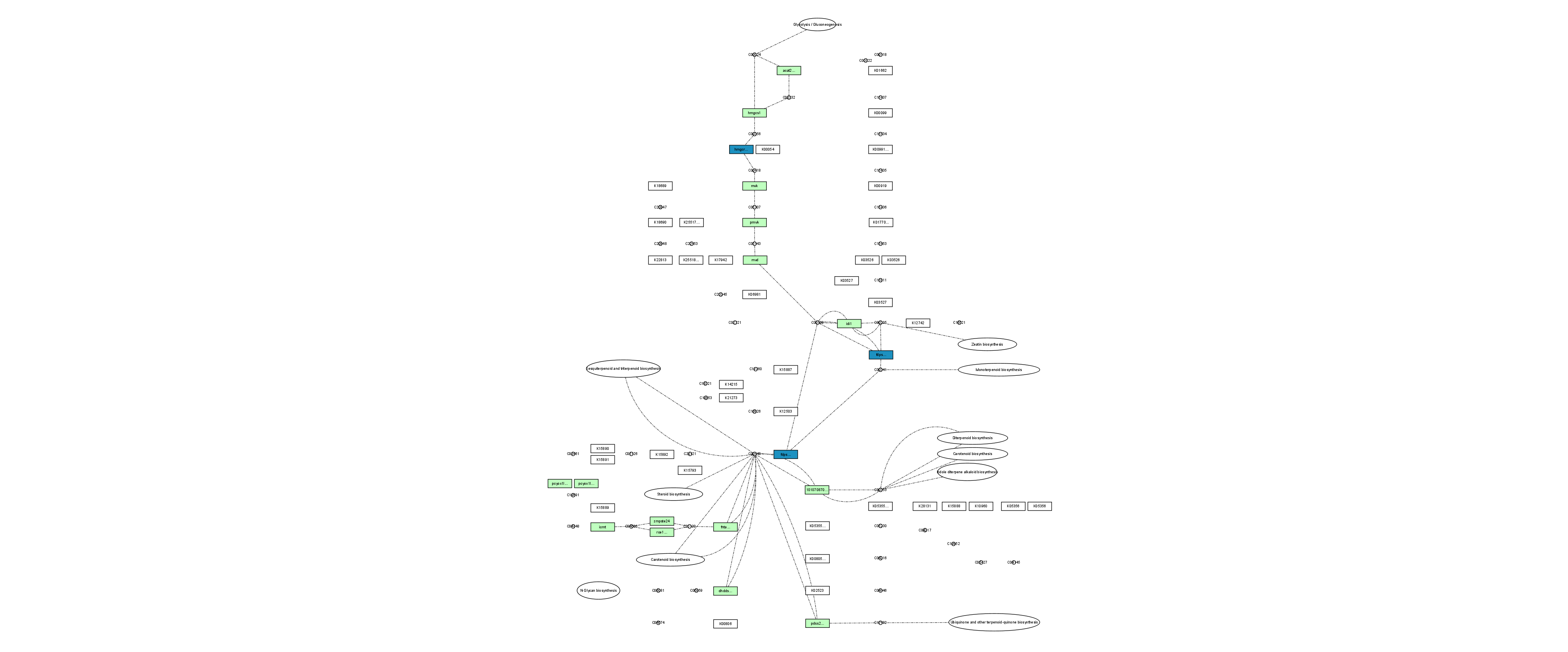

Supplement: Supplementary file 1 [file marinedrugs-24-00172-s001.zip › Supplementary figure/Figure S2/Terpenoid backbone biosynthesis.png]
